# 4-Bromo-2-chloro­aniline

**DOI:** 10.1107/S1600536809054944

**Published:** 2009-12-24

**Authors:** Zan-Bin Wei, Zhi-Hong Liu, Jian-Liang Ye, Hong-Kui Zhang

**Affiliations:** a The Key Laboratory for Chemical Biology of Fujian Province, College of Chemistry and Chemical Engineering, Xiamen University, Xiamen, Fujian 361005, People’s Republic of China

## Abstract

The title compound, C_6_H_5_BrClN, is almost planar (r.m.s. deviation = 0.018 Å). In the crystal, mol­ecules are linked by inter­molecular N—H⋯N and weak N—H⋯Br hydrogen bonds, generating sheets.

## Related literature

For background to halogentaed aromatic compounds, see: Katritzky *et al.* (1994 ▶). For related structures, see: Cox (2001 ▶); Parkin *et al.* (2005 ▶); Ng (2005 ▶); Ferguson *et al.* (1998 ▶). For the synthesis, see: Ault & Kraig (1966 ▶).
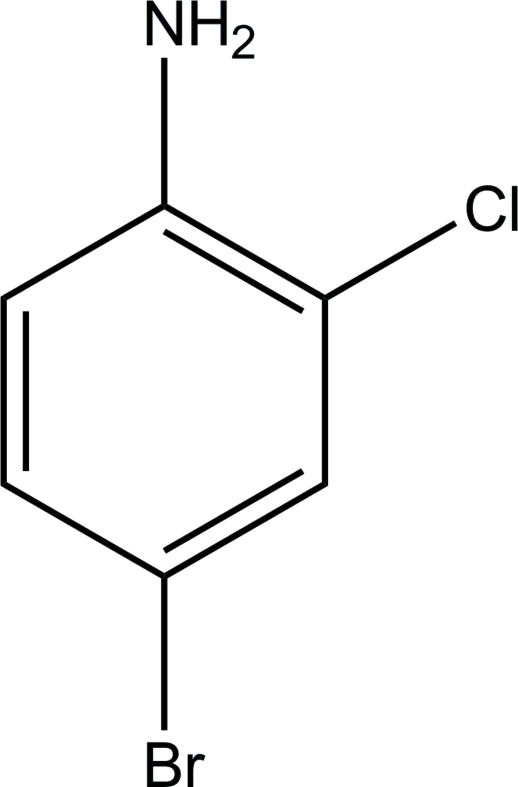


## Experimental

### 

#### Crystal data

C_6_H_5_BrClN
*M* *_r_* = 206.47
Orthorhombic, 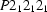

*a* = 10.965 (4) Å
*b* = 15.814 (6) Å
*c* = 4.0232 (15) Å
*V* = 697.7 (4) Å^3^
*Z* = 4
Mo *K*α radiation
μ = 6.17 mm^−1^
*T* = 298 K
0.7 × 0.19 × 0.15 mm

#### Data collection

Bruker SMART CCD diffractometer
Absorption correction: multi-scan (*SADABS*; Bruker, 2001 ▶) *T*_min_ = 0.254, *T*_max_ = 0.396
5799 measured reflections
1710 independent reflections
1333 reflections with *I* > 2σ(*I*)
*R*_int_ = 0.044

#### Refinement

*R*[*F* ^2^ > 2σ(*F* ^2^)] = 0.033
*wR*(*F* ^2^) = 0.081
*S* = 0.99
1710 reflections
83 parameters
H-atom parameters constrained
Δρ_max_ = 0.33 e Å^−3^
Δρ_min_ = −0.48 e Å^−3^
Absolute structure: Flack (1983 ▶), 511 Friedel pairs
Flack parameter: 0.035 (15)

### 

Data collection: *SMART* (Bruker, 2001 ▶); cell refinement: *SAINT* (Bruker, 2001 ▶); data reduction: *SAINT*; program(s) used to solve structure: *SHELXTL* (Sheldrick, 2008 ▶); program(s) used to refine structure: *SHELXTL*; molecular graphics: *ORTEP-3* (Farrugia, 1997 ▶); software used to prepare material for publication: *SHELXTL*.

## Supplementary Material

Crystal structure: contains datablocks I, global. DOI: 10.1107/S1600536809054944/hb5285sup1.cif

Structure factors: contains datablocks I. DOI: 10.1107/S1600536809054944/hb5285Isup2.hkl

Additional supplementary materials:  crystallographic information; 3D view; checkCIF report

## Figures and Tables

**Table 1:** Hydrogen-bond geometry (Å, °)

*D*—H⋯*A*	*D*—H	H⋯*A*	*D*⋯*A*	*D*—H⋯*A*
N1—H1*B*⋯Br1^i^	0.86	3.04	3.719 (3)	137
N1—H1*A*⋯N1^ii^	0.86	2.34	3.172 (4)	164

Symmetry codes: (i) 
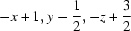
; (ii) 
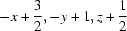
.

**Fig. 1.**
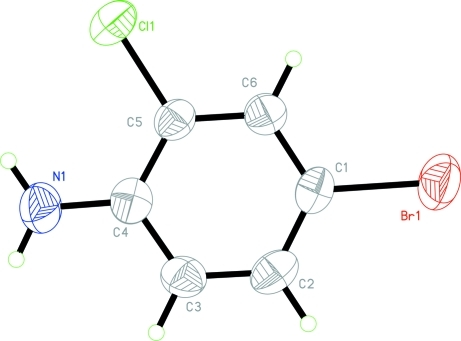
The molecular structure of (I) showing 50% probability displacement ellipsoids.

**Fig. 2.**
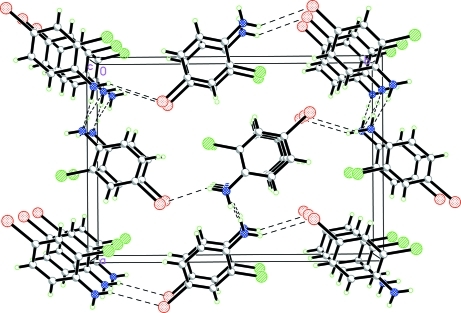
The packing of (I), viewed down the c axis. N—H···N and N—H···Br hydrogen bond interactions are shown as dashed lines.

## supplementary crystallographic information

### Comment

Halogenated aromatic compounds is an important class of intermediates for the synthesis of bio-active substances such as antibacterial, antioxidizing, antiviral agents (e.g. Katritzky *et al.*, 1994). Despite their simple structures, the X-ray structures of halogenated aniline compounds periodically were reported, such as 2,5-dichloroaniline (Cox, 2001), 2-iodoaniline (Parkin *et al.*, 2005) and 5-chloro-2-nitroaniline (Ng, 2005).  We now report the title compound, (I).

The packing of molecules in the crystal structure is stabilized and linked into a two-dimensional texture by intermolecular N—H···N and N—H···Br hydrogen bonds. The N···N distance is 3.172 (4) Å in hydrogen bond N—H···N, which are similar to that observed in 2,4-dibromo-6- chloroaniline (Ferguson *et al.*, 1998), 3.150 (11) Å and 2-iodoaniline (Parkin *et al.*, 2005), 3.161 (14) Å.

### Experimental

The tiltle compound was prepared according to a previously reported method (Ault & Kraig, 1966). Colourless needles of (I) were obtained by slow evaporation of a petroleum ether solution.

### Refinement

The hydrogen atoms were positioned geometrically, with C—H = 0.93, 0.98, 0.97 and 0.96 Å for phenyl, methine, methylene and methyl H atoms, respectively, and were included in the refinement in the riding model approximation. The displacement parameters of methyl H atoms were set to 1.5*U*_eq_(C), while those of other H atoms were set to 1.2*U*_eq_(C). In the absence of significant anomalous scattering effects, Friedel pairs were merged.

### Crystal data

**Table:**

C_6_H_5_BrClN	*F*(000) = 424
*M**_r_* = 206.47	*D*_x_ = 1.965 Mg m^−^^3^
Orthorhombic, *P*2_1_2_1_2_1_	Mo *K*α radiation, λ = 0.71073 Å
Hall symbol: P 2ac 2ab	Cell parameters from 1199 reflections
*a* = 10.965 (4) Å	θ = 2.3–29.8°
*b* = 15.814 (6) Å	µ = 6.17 mm^−^^1^
*c* = 4.0232 (15) Å	*T* = 298 K
*V* = 697.7 (4) Å^3^	Needle, colourless
*Z* = 4	0.7 × 0.19 × 0.15 mm

### Data collection

**Table:**

Bruker SMART CCD diffractometer	1710 independent reflections
Radiation source: fine-focus sealed tube	1333 reflections with *I* > 2σ(*I*)
graphite	*R*_int_ = 0.044
φ and ω scan	θ_max_ = 29.8°, θ_min_ = 2.3°
Absorption correction: multi-scan (*SADABS*; Bruker, 2001)	*h* = −14→14
*T*_min_ = 0.254, *T*_max_ = 0.396	*k* = −20→21
5799 measured reflections	*l* = −5→5

### Refinement

**Table:**

Refinement on *F*^2^	Hydrogen site location: inferred from neighbouring sites
Least-squares matrix: full	H-atom parameters constrained
*R*[*F*^2^ > 2σ(*F*^2^)] = 0.033	*w* = 1/[σ^2^(*F*_o_^2^) + (0.0374*P*)^2^] where *P* = (*F*_o_^2^ + 2*F*_c_^2^)/3
*wR*(*F*^2^) = 0.081	(Δ/σ)_max_ = 0.008
*S* = 0.99	Δρ_max_ = 0.33 e Å^−^^3^
1710 reflections	Δρ_min_ = −0.48 e Å^−^^3^
83 parameters	Extinction correction: *SHELXTL* (Sheldrick, 2008), Fc^*^=kFc[1+0.001xFc^2^λ^3^/sin(2θ)]^-1/4^
0 restraints	Extinction coefficient: 0.246 (8)
Primary atom site location: structure-invariant direct methods	Absolute structure: Flack (1983), 511 Friedel pairs
Secondary atom site location: difference Fourier map	Flack parameter: 0.035 (15)

### Special details

**Table:**

Geometry. All esds (except the esd in the dihedral angle between two l.s. planes) are estimated using the full covariance matrix. The cell esds are taken into account individually in the estimation of esds in distances, angles and torsion angles; correlations between esds in cell parameters are only used when they are defined by crystal symmetry. An approximate (isotropic) treatment of cell esds is used for estimating esds involving l.s. planes.
Refinement. Refinement of F^2^ against ALL reflections. The weighted R-factor wR and goodness of fit S are based on F^2^, conventional R-factors R are based on F, with F set to zero for negative F^2^. The threshold expression of F^2^ > 2sigma(F^2^) is used only for calculating R-factors(gt) etc. and is not relevant to the choice of reflections for refinement. R-factors based on F^2^ are statistically about twice as large as those based on F, and R- factors based on ALL data will be even larger.

### Fractional atomic coordinates and isotropic or equivalent isotropic displacement parameters (Å^2^)

**Table:**

	*x*	*y*	*z*	*U*_iso_*/*U*_eq_	
Br1	0.28337 (3)	0.74386 (2)	0.51936 (10)	0.0646 (2)	
Cl1	0.42519 (8)	0.41363 (5)	0.4648 (3)	0.0588 (3)	
C1	0.3957 (3)	0.65802 (18)	0.6258 (8)	0.0432 (7)	
C2	0.4999 (3)	0.6770 (2)	0.7973 (9)	0.0494 (8)	
H2A	0.5151	0.7322	0.8656	0.059*	
C3	0.5812 (3)	0.6145 (2)	0.8673 (9)	0.0456 (8)	
H3A	0.6518	0.6276	0.9846	0.055*	
C4	0.5613 (3)	0.5320 (2)	0.7681 (8)	0.0428 (8)	
C5	0.4548 (3)	0.51544 (19)	0.5957 (8)	0.0395 (7)	
C6	0.3728 (2)	0.57746 (17)	0.5249 (7)	0.0431 (7)	
H6A	0.3018	0.5649	0.4087	0.052*	
N1	0.6464 (2)	0.47087 (18)	0.8346 (8)	0.0565 (8)	
H1A	0.7124	0.4838	0.9383	0.068*	
H1B	0.6338	0.4196	0.7726	0.068*	

### Atomic displacement parameters (Å^2^)

**Table:**

	*U*^11^	*U*^22^	*U*^33^	*U*^12^	*U*^13^	*U*^23^
Br1	0.0770 (3)	0.0451 (2)	0.0717 (3)	0.01789 (15)	−0.0064 (2)	0.0025 (2)
Cl1	0.0638 (5)	0.0349 (4)	0.0776 (6)	−0.0034 (3)	−0.0023 (5)	−0.0047 (5)
C1	0.0509 (17)	0.0347 (16)	0.0438 (16)	0.0061 (14)	0.0043 (14)	0.0027 (13)
C2	0.0582 (18)	0.0357 (18)	0.054 (2)	−0.0081 (15)	0.0075 (16)	−0.0027 (15)
C3	0.0368 (15)	0.0481 (19)	0.0520 (18)	−0.0064 (14)	−0.0002 (14)	−0.0008 (15)
C4	0.0399 (16)	0.0430 (18)	0.0455 (18)	0.0005 (14)	0.0086 (14)	0.0040 (13)
C5	0.0416 (15)	0.0344 (15)	0.0424 (16)	−0.0028 (12)	0.0053 (13)	0.0012 (12)
C6	0.0424 (14)	0.0406 (15)	0.0461 (16)	−0.0022 (12)	−0.0007 (16)	0.0016 (16)
N1	0.0434 (15)	0.0500 (17)	0.076 (2)	0.0101 (13)	−0.0015 (15)	0.0006 (15)

### Geometric parameters (Å, °)

**Table:**

Br1—C1	1.883 (3)	C3—H3A	0.9300
Cl1—C5	1.725 (3)	C4—N1	1.369 (4)
C1—C6	1.361 (4)	C4—C5	1.383 (4)
C1—C2	1.368 (5)	C5—C6	1.361 (4)
C2—C3	1.361 (5)	C6—H6A	0.9300
C2—H2A	0.9300	N1—H1A	0.8600
C3—C4	1.382 (4)	N1—H1B	0.8600
			
C6—C1—C2	120.7 (3)	C3—C4—C5	117.1 (3)
C6—C1—Br1	119.1 (2)	C6—C5—C4	121.8 (3)
C2—C1—Br1	120.2 (2)	C6—C5—Cl1	119.0 (2)
C3—C2—C1	119.4 (3)	C4—C5—Cl1	119.2 (2)
C3—C2—H2A	120.3	C1—C6—C5	119.4 (3)
C1—C2—H2A	120.3	C1—C6—H6A	120.3
C2—C3—C4	121.6 (3)	C5—C6—H6A	120.3
C2—C3—H3A	119.2	C4—N1—H1A	120.0
C4—C3—H3A	119.2	C4—N1—H1B	120.0
N1—C4—C3	120.2 (3)	H1A—N1—H1B	120.0
N1—C4—C5	122.7 (3)		

### Hydrogen-bond geometry (Å, °)

**Table:**

*D*—H···*A*	*D*—H	H···*A*	*D*···*A*	*D*—H···*A*
N1—H1B···Br1^i^	0.86	3.04	3.719 (3)	137
N1—H1A···N1^ii^	0.86	2.34	3.172 (4)	164

Symmetry codes: (i) −*x*+1, *y*−1/2, −*z*+3/2; (ii) −*x*+3/2, −*y*+1, *z*+1/2.
